# Monoclonal Gammopathy Prevalence in Newly Diagnosed Prostate Cancer Patients: A Correlative Perspective Observational Study

**DOI:** 10.3390/cancers17233790

**Published:** 2025-11-27

**Authors:** Gabriele Tulone, Nicola Pavan, Rosa Giaimo, Anna Martorana, Giuseppe Salvaggio, Giuseppe Cutaia, Francesco Claps, Emilia Gigliotta, Dalila Marmo, Cristina Minasola, Sergio Siragusa, Alchiede Simonato, Cirino Botta

**Affiliations:** 1 Department of Precision Medicine in the Medical, Surgical and Critical Care Area (Me.Pre.C.C.), University of Palermo, 90141 Palermo, Italy; nicola.pavan@unipa.it (N.P.); rosa.giaimo@policlinico.pa.it (R.G.); cristinaminasola@gmail.com (C.M.); alchiede.simonato@unipa.it (A.S.); 2 Department of Health Promotion, Mother and Child Care, Internal Medicine and Medical Specialties (ProMISE), University of Palermo, 90141 Palermo, Italy; anna.martorana@unipa.it (A.M.); emiliagigliotta@unipa.it (E.G.); dalila.marmo@community.unipa.it (D.M.); sergio.siragusa@unipa.it (S.S.); 3 Section of Radiology, BiND, University Hospital “Paolo Giaccone”, University of Palermo, 90127 Palermo, Italy; giuseppe.salvaggio@policlinico.pa.it (G.S.); cutaiagiuseppe7@gmail.com (G.C.); 4 Oncological Urology, Veneto Institute of Oncology IRCCS, via Gattamelata 64, 35128 Padua, Italy; claps.francesco@gmail.com

**Keywords:** MGUS, prostate cancer, prostate biopsy

## Abstract

Background: While there have been limited reports suggesting a possible association between Monoclonal Gammopathy (MG) of Undetermined Significance (MGUS) and prostate cancer, a clear biological correlation has yet to be established. Methods: In this study, we aimed to investigate the incidence of MGUS in a cohort of 168 patients undergoing TRUSBx for suspected prostate cancer between September 2022 and December 2023. Results: Our findings revealed that the incidence of MGUS, identified by serum immunofixation or abnormal free light chain ratio, was significantly higher in this cohort than the anticipated global incidence (33.93% vs. 5%). Furthermore, the prevalence of MGUS was higher in patients with a Gleason Score (GS) exceeding 7 compared to those with GS6 or with ASAP/HG PIN (34.2% vs. 28% vs. 25%, respectively). A systematic univariate analysis of 42 clinical and biological variables identified fibrinogen levels, neutrophil-to-lymphocyte ratio, and the percentage of alpha2 band in serum protein electrophoresis as significantly associated with MGUS presence. Conclusions: These findings suggest that a systemic inflammatory status and a highest GS in these patients may increase the likelihood of detecting an MGUS. To our knowledge, this study is the first to suggest an association between prostate cancer and MG. Further and larger studies are required to confirm the increased prevalence of MGUS within this target population and to establish the clinical relevance of these precocious diagnoses.

## Simple Summary

Monoclonal gammopathy of undetermined significance (MGUS) is a common plasma-cell disorder in the elderly and is typically considered a precursor of multiple myeloma. However, its relationship with solid tumors, including prostate cancer, remains poorly understood. In this study, we evaluated the prevalence of MGUS among 168 men undergoing prostate biopsy for suspected prostate cancer. We found that MGUS was present in about one-third of the patients—significantly higher than expected for the general population. The occurrence of MGUS was more frequent in individuals with higher Gleason scores and in those showing signs of systemic inflammation, such as increased fibrinogen levels and a higher neutrophil-to-lymphocyte ratio. These findings suggest that prostate inflammation and cancer may share biological pathways with monoclonal gammopathies, possibly through chronic immune activation and inflammatory microenvironmental changes. This work highlights the importance of recognizing MGUS in patients with prostate pathology, as its detection may offer new insights into the interplay between systemic inflammation, prostate cancer progression, and plasma cell disorders.

## 1. Introduction

### 1.1. Prostate Cancer (PCa)

PCa is the second most common malignancy among elderly men, with 1.2 million cases worldwide in 2020, and the fifth leading cause of cancer-related mortality [1,2]. Nearly all patients with localized PCa achieve a five-year survival [3,4]. Both genetic and environmental factors contribute to PCa risk. Family history and ethnic background strongly influence incidence, while hereditary prostate cancer (HPCa) is rare but associated with earlier onset [5,6,7,8]. Genome-wide association studies have identified more than 100 susceptibility loci, particularly involving BRCA2, CHEK2, ATM, and BRCA1, with clinical implications for genetic testing and targeted therapy [9,10,11]. Environmental influences include diet, obesity, smoking, alcohol and coffee intake, inflammatory bowel disease, and HPV-16 infection, all of which may affect incidence and progression [12,13,14]. Lifestyle factors such as physical and sexual activity may be protective, and a meta-analysis reported a modest reduction in PCa risk with circumcision [15].

### 1.2. Monoclonal Gammopathy of Undetermined Significance (MGUS)

Monoclonal gammopathy of undetermined significance (MGUS) is an age-related premalignant clonal plasma-cell disorder, with a prevalence of approximately 5% in individuals older than 50 years. Contemporary guidance emphasizes confirming the presence of a monoclonal protein by serum protein electrophoresis and immunofixation/immunosubtraction, assessing free light chains, and excluding CRAB features (hypercalcemia, renal insufficiency, anemia, and bone lesions) of overt myeloma [16,17,18]. Beyond its role as a precursor to hematological malignancies, recent studies have suggested associations between MGUS and solid tumors. A population-based analysis reported a 10% increased risk of MGUS among individuals with prior cancers, particularly hepatic, biliary, pancreatic, and urinary tract malignancies [19]. An abstract from *Blood* 2024 [20] described an increased incidence of solid tumors, including prostate cancer, in patients with MGUS. Conversely, a large cohort of 14,626 patients with solid tumors found a higher prevalence of M-protein in those with lung cancer (3.7% vs. 2.2%, *p* < 0.001), but not in other tumor types [21]. Collectively, these findings highlight the need to clarify whether MGUS is a coincidental finding in cancer patients or reflects shared biological mechanisms linking clonal plasma-cell expansion and tumorigenesis. While its relationship with prostate cancer (PCa) remains uncertain, MGUS-related immune dysregulation and increased cancer susceptibility warrant further investigation [22]. By definition, MGUS is characterized by a serum monoclonal protein concentration ≤3.0 g/dL, absent or minimal monoclonal light chains in the urine, bone marrow infiltration ≤10% monoclonal plasma cells, and no CRAB features [23,24]. Evidence linking MGUS to prostate pathology remains limited, with most reports confined to single cases [25]. Here, we aimed to determine the prevalence of MGUS among men undergoing prostate biopsy for suspected PCa, and to explore whether clinical or biochemical features, including inflammatory markers, are associated with MGUS detection.

## 2. Materials and Methods

### 2.1. Samples Collection

We conducted this analysis within the observational monocentric prospective and retrospective study MMVision (approved by our internal ethical committee with the number 02/2022). We prospectively enrolled all men undergoing transrectal or transperineal ultrasound-guided prostate biopsy between September 2022 and December 2023 at our institution. Prostate biopsies were performed following European guidelines on antibiotic prophylaxis [26].

All participants were biopsy-naïve and referred for suspected prostate cancer based on abnormal PSA and/or multiparametric MRI findings.

In addition to demographic and clinical data, we systematically recorded prior or concurrent malignancies, categorized as hematological, urological, gastrointestinal, or other solid tumors. Comorbid conditions were also extracted from medical history, including cardiovascular disease, hypertension, diabetes mellitus, chronic kidney disease, chronic liver disease, and chronic obstructive pulmonary disease. For statistical analysis, comorbidities and prior/concurrent malignancy status were evaluated as categorical variables. Associations with the presence of MGUS were tested using chi-square or Fisher’s exact test as appropriate. Variables with *p* < 0.10 in univariate analysis were further entered into a multivariable logistic regression model to adjust for potential confounding factors such as age and PSA. Results are reported as odds ratios (OR) with 95% confidence intervals (CI).

Data were collected from 168 consecutive patients undergoing transrectal ultrasound-guided prostate biopsy (TRUSBx) due to a strong suspicion of prostate cancer (PCa) based on elevated Prostate-Specific Antigen (PSA) levels and abnormal digital rectal examination (DRE) findings. All patients underwent multiparametric magnetic resonance imaging (mpMRI) prior to biopsy, following established clinical guidelines. A minimum of 12 core samples were obtained per patient, with additional targeted biopsies performed in cases where mpMRI detected lesions. Histological results, summarized in Table 1, were analyzed by classifying patients into benign and malignant groups. Malignant cases were further subclassified based on Gleason Score (GS). To ensure the representativeness of our sample, we assessed the association of known variables (e.g., PSA levels and age) with GS. We investigated the incidence rate of MGUS within this cohort using serum protein electrophoresis (SPE), serum immunosubtraction on capillary electrophoresis, immunofixation electrophoresis (IFE), and serum free light chain (FLC) assays. These diagnostic and monitoring tests for monoclonal gammopathies (MG) are routinely performed in our institution. All patients diagnosed with MGUS or multiple myeloma were subsequently followed up at the hematology outpatient clinic for further evaluation and management, and classified into risk classes according to immunoglobulin isotype, M-protein abundance, and alterations of the FLC ratio [23].

### 2.2. Staining Procedures

Immunohistochemistry was performed on paraffin-embedded prostate tissue sections, using anti-CD138 (clone MI15, Leica Biosystems, Nussloch, Germany), anti-CD20 (clone L25, Leica Biosystems), and anti-CD3 (clone LN10, Leica Biosystems) antibodies. In addition, kappa and lambda light chains were evaluated on the slides to respectively identify plasma cells, B cells, and T cells. Immune infiltrating cells were than evaluated by 2 different pathologists and mean values were considered for further statistical analyses.

### 2.3. Statistical Analysis

All statistical analyses were performed using R software (version 4.3.2; R Foundation for Statistical Computing, Vienna, Austria). Continuous variables were assessed for normality using the Shapiro–Wilk test. Normally distributed variables were summarized as mean ± standard deviation (SD), while non-normally distributed variables were expressed as median and interquartile range (IQR). Categorical variables were presented as absolute counts and percentages.

Comparisons between two groups were conducted using the Student’s *t*-test for normally distributed variables and the Mann–Whitney U test for non-normally distributed variables. Categorical variables were compared using the Chi-square test or Fisher’s exact test, as appropriate. Univariate analyses were carried out to evaluate the association between clinical, biochemical, and histopathological parameters and the presence of MGUS. A GLM was performed as a multivariate model and FDR correction for multiple testing.

Correlations between continuous variables were assessed using Pearson or Spearman correlation coefficients, depending on the data distribution. For visualization, correlation matrices, heatmaps, and boxplots were generated using the ggplot2, ggpubr, complexheatmaps, and corrplot R packages (R 4.3.2). All statistical tests were two-tailed, and a *p* value < 0.05 was considered statistically significant.

## 3. Results

### 3.1. Patients’ Characteristics

**Figure 1:**
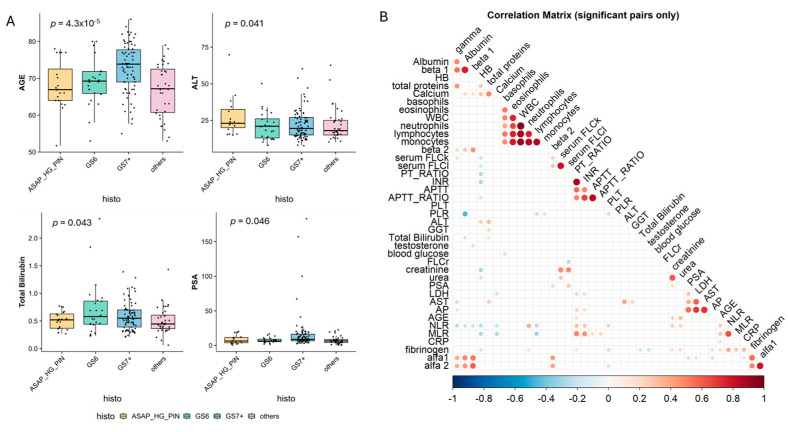
(**A**) Boxplot reporting clinical and laboratory parameters significantly associated with histological conditions. (**B**) Correlation plot showing the presence of significant correlations among variables. The deeper/stronger the color, the stronger the correlation between the two variables.

The study evaluated 168 patients, all of whom underwent prostate biopsy for the first time. A total of 2156 prostate tissue cores were examined, obtained via transrectal or transperineal prostate biopsy. The median patient age was 70 years (range: 51–85 years), with a median PSA level of 7.73 ng/mL (IQR 7.16). Additionally, significant differences were observed in selected clinical and biochemical parameters according to histological classification (Figure 1A). Patients with GS7+ lesions were significantly older compared to those with ASAP/HG-PIN or GS6 histology (*p* = 4.3 × 10^−5^), and showed higher ALT levels (*p* = 0.041). Total bilirubin and PSA values also varied across histological groups (*p* = 0.043 and *p* = 0.046, respectively). The correlation matrix (Figure 1B) highlights the main relationships among laboratory parameters. Strong positive correlations were observed among liver function tests (ALT, AST, GGT, total bilirubin), between inflammatory markers (CRP, NLR, fibrinogen, *α*-globulins), and among protein fractions (albumin, *β*- and *γ*-globulins). Conversely, several of these variables were negatively correlated with hematologic indices and renal function markers. Together, these findings indicate coordinated variations in metabolic and inflammatory profiles across histological categories, suggesting that biochemical alterations may parallel the degree of tissue transformation.

**Figure 2:**
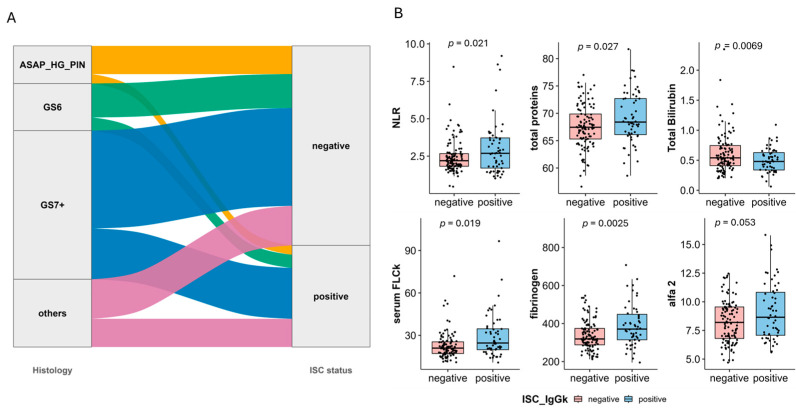
(**A**) Sankey plot reporting the distribution of ISC positivity according to histological condition; (**B**) Boxplot representing the 6 parameters which significantly differs among the 2 subgroups (MGUS vs. not MGUS) with *p* values obtained through Student’s T test.

**Figure A1:**
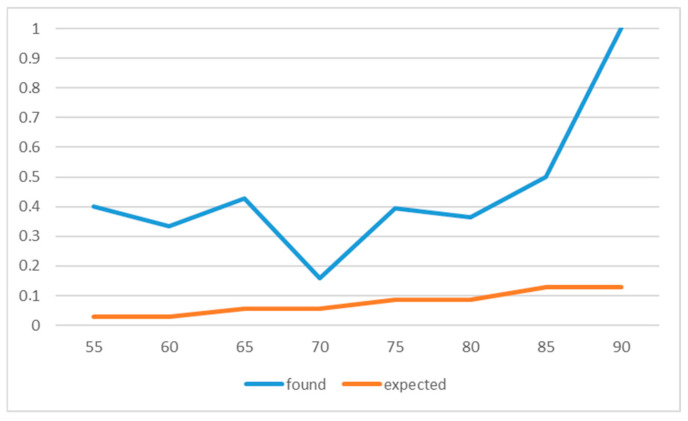
Comparison between expected MGUS incidence curves (according to age), and what we found in our cohort.

As reported in Table 1, 57 patients (33.93%) exhibited positive IFE/LC results, with almost all of them classified as low-risk MGUS. This prevalence significantly exceeded the anticipated global incidence of MGUS (Appendix A: Figure A1), and 33.93% is significantly higher than expected 5% (*p* < 0.001). Notably, as illustrated in Figure 2A, MGUS prevalence was higher in patients with a GS > 7 than in those with GS6 or ASAP/HG PIN (34.2% vs. 28% vs. 25%, respectively) (Figure 2A,B).

### 3.2. Biochemical Predictors of MGUS

**Figure A2:**
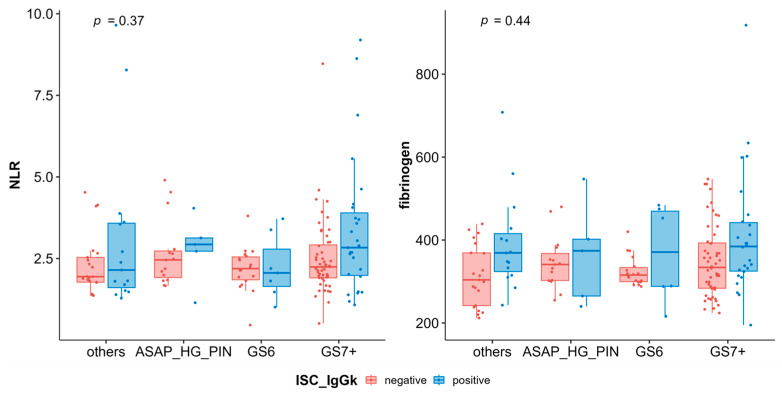
Boxplot demonstrating the absence of correlation between histological grade and inflammatory markers.

To identify potential factors linked to MGUS, we conducted a systematic univariate analysis of 42 clinical and biological variables. The analysis highlighted the following significant associations with MGUS presence: neutrophil-to-lymphocyte ratio (NLR), fibrinogen levels, percentage of the alpha-2 band in serum protein electrophoresis. Conversely, higher total bilirubin levels were linked to the absence of MGUS (Appendix A: Table A1 and Figure 1B). Applying a generalized linear model for multivariate analysis, both fibrinogen and NLR remained significantly associated with MGUS presence (data not shown). Additionally, as expected, total protein levels and an elevated free light chain ratio (FLCr) were strongly associated with MGUS presence. Interestingly, only a few weak but significant correlations were found among the identified variables (Appendix A: Figure A2), suggesting that a systemic inflammatory status in these patients might increase the likelihood of detecting an M-component. Further analysis investigated whether the increased MGUS prevalence was age-related. However, the incidence remained relatively stable when compared to the general population. This supports the hypothesis that prostate inflammation and/or cancer, rather than age, might be the underlying factor driving MGUS prevalence (Appendix A: Figure A2). Additional parameters were also evaluated, including liver function markers, renal function markers, serum electrolytes, coagulation indices, and LDH levels. None of these parameters demonstrated statistically significant differences between the study populations. These findings highlight a potential link between systemic inflammation, prostate pathology, and MGUS prevalence, warranting further investigation into the underlying mechanisms connecting these conditions.

In the complete-case analysis set (*n* = 104), MGUS was detected in 52/168 patients (30.9%) (Appendix A: Figure A2). Given the elevated *α*2-globulin levels observed in prostate cancer (PCa) patients, we performed a multivariable logistic regression including *α*2-globulin fraction, PCa status, neutrophil-to-lymphocyte ratio (NLR), fibrinogen, age, and PSA. Adjusted associations with MGUS were as follows: *α*2-globulin (OR 1.12, 95% CI 0.83–1.50, *p* = 0.47), PCa status (OR 1.99, 95% CI 0.75–5.28, *p* = 0.17), NLR (OR 1.28 per unit, 95% CI 0.95–1.73, *p* = 0.10), fibrinogen (OR 1.004 per mg/dL, 95% CI 0.999–1.010, *p* = 0.14), age (OR 1.02 per year, 95% CI 0.96–1.09, *p* = 0.47), and PSA (OR 0.97 per ng/mL, 95% CI 0.92–1.03, *p* = 0.33). None of these predictors reached statistical significance, although trends were observed for PCa and NLR (Appendix A: Table A2 and Table A3).

### 3.3. Prostate Microenvironment Associated with MGUS Incidence

**Figure 3:**
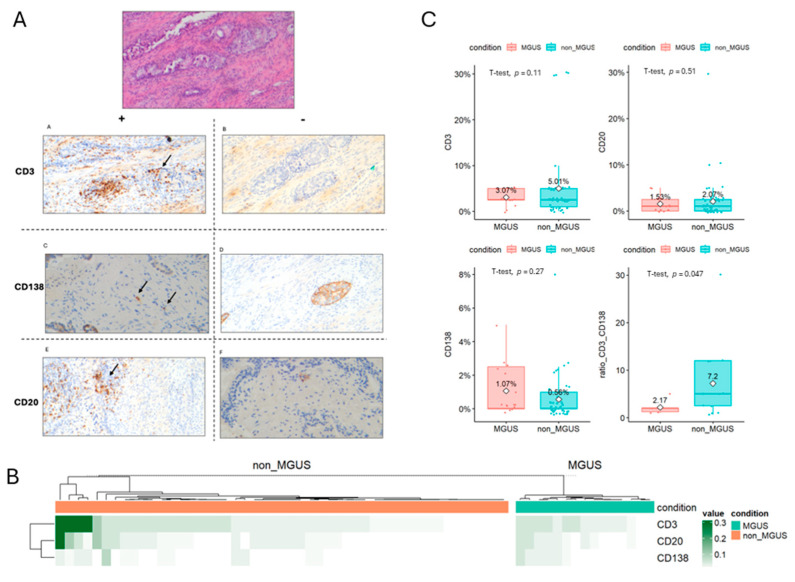
Prostatic adenocarcinoma and tumor microenvironment. In (**A**) Representative staining for Hematoxylin and Eosin staining (10× magnification), and negative/positive staining for CD3, CD138, and CD20. In (**B**) Heatmap reporting the differences of cell infiltration in each patient grouped according to MGUS or non-MGUS status. In (**C**) Boxplot reporting different immune microenvironment cells infiltration in tumor microenvironment.

To evaluate the potential association between the prostate microenvironment and MGUS susceptibility, we performed an immunohistochemical quantification of CD3 (T cells), CD20 (B cells), and CD138 (plasma cells) in a subset of 64 patients (from a total cohort of 168) for whom formalin-fixed paraffin-embedded prostate tissues were available (Figure 3A). Patients were categorized according to the presence or absence of monoclonal gammopathy (Figure 3B). CD3+ lymphocyte infiltration showed no significant difference between MGUS and non-MGUS patients, even if a trend with an increased infiltration in non-MGUS patients had been observed (median 3.07% vs. 5.01% respectively, *p* = 0.11). No differences were observed for CD20+ B cell infiltration (median 1.53% vs. 2.7% respectively, *p* = 0.51), while a non-significant trend (*p*: 0.27) was observed for plasma cell infiltration (never restricted for light chain, i.e., polyclonal), being higher in MGUS subjects as compared to non-MGUS patients (median 1.07% vs. 0.56%). When evaluable, the ratio between T cells and plasma cells was significantly higher for patients in the non-MGUS group, supporting a potential reduction in T cell control in favor of “myeloid” inflammation. Indeed, these findings suggest that a polyclonal plasma cell infiltration (increased inflammation?) in the presence of a reduced T cell activation tends to be higher in MGUS subjects as compared-to non-MGUS cases, in line with inflammatory-based disease pathobiology (Figure 3).

## 4. Discussion

Our study is the first large-scale investigation correlating MGUS with a specific cancer risk group, providing valuable evidence that prostate pathology—both benign and malignant—may be linked to an increased prevalence of MGUS. Consistent with our findings, prior case reports [27,28] have described isolated instances of metastatic prostate cancer coexisting with MGUS, suggesting an inflammation-driven link between these conditions. Advanced malignancies are known to trigger chronic immune activation and systemic inflammation, which in turn can contribute to clonal plasma cell expansion and MGUS development. This aligns with well-established knowledge that inflammation plays a fundamental role in both prostate cancer progression and plasma cell dyscrasias. Chronic inflammation in the tumor microenvironment can lead to increased cytokine production (e.g., IL-6, TNF-*α*, IL-1*β*), which promotes both prostate cancer progression and the survival/proliferation of abnormal plasma cell clones in MGUS [29,30]. Altered immune surveillance may further facilitate both the persistence of neoplastic prostate cells and the emergence of monoclonal gammopathies. A pro-tumorigenic microenvironment, where oxidative stress, chronic antigenic stimulation, and DNA damage create favorable conditions for both prostate cancer progression and clonal plasma cell expansion. Unlike prior studies that mainly focused on advanced cancers, our data reveal that MGUS can already be detected in early-stage prostate disease. Unlike prior studies that focused primarily on advanced-stage malignancies, our findings demonstrate a potential early association between MGUS and prostate pathology, irrespective of overt metastases. Interestingly, our results align with recent findings from the iSTOPmm study, which identified an increased risk of MG in cancer patients, particularly those with urinary tract malignancies [31]. However, there is a key distinction; the iSTOPmm study was limited to patients already diagnosed with advanced-stage cancer, whereas our study examined a broader population at high risk for prostate cancer, including those with early-stage disease. This suggests that the MGUS–prostate cancer association is not confined to metastatic disease but may also be relevant in earlier cancer stages, especially in cases characterized by high inflammatory activity and higher Gleason scores (GS) (Appendix A: Figure A2). Our findings raise important questions regarding the clinical significance of MGUS detection in patients with prostate pathology. MGUS could serve as a biomarker of systemic inflammation in prostate cancer. Patients with both MGUS and prostate cancer undergo closer surveillance for hematologic malignancies. Furthermore, patients with MGUS might benefit from closer surveillance for prostate malignancies, given the potential association between the two conditions. Conversely, patients with prostate pathology—particularly those with high-grade tumors or significant inflammation—may warrant further evaluation for the presence of MGUS, as early detection could have important implications for both hematologic and oncologic monitoring. Supposing that the inflammatory milieu in the prostate influences MGUS progression, or vice versa, it becomes crucial to explore the bidirectional relationship between these conditions. Chronic inflammation is known to play a pivotal role in both prostate carcinogenesis and the expansion of clonal plasma cells, potentially serving as a common driver of disease progression, as further demonstrated in extramedullary lesions from MM [19]. While our data suggest a potentially meaningful association, further research is needed to dissect the underlying mechanisms, particularly by investigating shared inflammatory and immune pathways. Longitudinal studies assessing the evolution of MGUS in patients with prostate pathology—and vice versa—could help determine whether prostate inflammation acts as a trigger for MGUS progression or whether MGUS-related immune dysregulation contributes to prostate disease onset or severity. Understanding these connections could open new avenues for targeted surveillance strategies and potential therapeutic interventions in both conditions. A larger, multicenter prospective study could provide more definitive insights into whether MGUS is simply a bystander in prostate cancer or an integral part of its inflammatory landscape. In summary, our findings highlight an emerging link between MGUS and prostate cancer, particularly in patients with high-grade or highly inflamed disease, suggesting that the two conditions may share common immuno-inflammatory drivers. This novel association underscores the importance of further investigating the interplay between prostate pathology, chronic inflammation, and plasma cell dyscrasias, which may have critical implications for cancer surveillance, risk stratification, and patient management. The *α*2-globulin fraction comprises several acute-phase proteins, including *α*2-macroglobulin, ceruloplasmin, and haptoglobin. These proteins are frequently elevated in conditions associated with systemic inflammation, liver disease, and certain types of anemia [32]. Their increase is therefore often interpreted as a non-specific marker of inflammatory or metabolic stress. In our cohort, elevated *α*2-globulin levels were observed in prostate cancer patients; however, after adjustment for inflammatory markers (neutrophil-to-lymphocyte ratio and fibrinogen), age, PSA, and biopsy results, *α*2-globulin was not independently associated with MGUS. This suggests that *α*2 elevation alone is unlikely to account for the presence of MGUS and may instead reflect underlying inflammation. Previous studies have reported similar associations, where *α*2-globulin fractions were linked with chronic disease states but not consistently with MGUS or cancer-specific risk [33].

## 5. Conclusions

Further biological and validation studies are needed to confirm the increased prevalence of MGUS within the target population, to deeply investigate the etiopathogenesis of this phenomenon, and to establish the clinical relevance of these early diagnoses, fostering a robust collaboration between urologists and hematologists for patient management.

## Figures and Tables

**Table 1:** Histopathological characteristics of the study cohort. Patient characteristics and distribution of MGUS according to histological findings.

Patients Characteristics	
Histological report	ASAP/HG PIN: 20 GS6: 25 GS7+: 79 Others/no cancer: 36 Missing: 8
MGUS	yes: 57 no: 111
MGUS patients characteristics	
Isotype	IgG *κ*: 44 IgG *λ*: 7 IgM *κ*: 1 IgM *λ*: 1 LC *κ*: 2 LC *λ*: 2
Risk class	Low Risk: 51 Int-low Risk: 6

Abbreviations: ASAP = atypical small acinar proliferation; HG PIN = high-grade prostatic intraepithelial neoplasia; GS = Gleason score; MGUS = monoclonal gammopathy of undetermined significance; IgG κ = immunoglobulin G kappa; IgG λ = immunoglobulin G lambda; IgM κ = immunoglobulin M kappa; IgM λ = immunoglobulin M lambda; LC κ = light chain kappa; LC λ = light chain lambda; Int-low risk = intermediate-low risk MGUS.

## Data Availability

The datasets generated and analyzed during the current study are not publicly available due to patient confidentiality and institutional ethical restrictions but are available from the corresponding author on reasonable request and subject to ethics approval.

## Appendix A

**Table A1:** Patients characteristics.

Variable	ISC Negative	ISC Positive	*p*-Val
AGE	69.84 (68.58–71.09)	71.32 (69.17–73.48)	0.24
WBC	7.50 (6.56–8.44)	8.19 (7.47–8.90)	0.25
neutrophils	4.62 (4.05–5.19)	5.31 (4.69–5.92)	0.11
lymphocytes	2.04 (1.76–2.33)	2.01 (1.79–2.24)	0.87
monocytes	0.61 (0.54–0.69)	0.63 (0.58–0.67)	0.71
eosinophils	0.26 (0.10–0.43)	0.20 (0.16–0.24)	0.46
basophils	0.04 (0.04–0.05)	0.05 (0.04–0.06)	0.32
HB	14.54 (14.28–14.80)	14.31 (13.86–14.77)	0.39
PLT	223.74 (212.19–235.28)	248.51 (222.34–274.68)	0.09
creatinine	1.01 (0.97–1.06)	1.07 (0.96–1.18)	0.33
total proteins	67.58 (66.83–68.32)	69.21 (67.96–70.46)	0.03
blood glucose	102.45 (97.54–107.37)	107.20 (100.53–113.87)	0.26
urea	42.26 (40.19–44.33)	44.02 (39.43–48.61)	0.49
AST	21.83 (19.61–24.05)	23.33 (21.00–25.67)	0.35
ALT	22.29 (20.04–24.54)	23.54 (20.28–26.80)	0.53
AP	96.84 (58.55–135.12)	78.32 (70.58–86.06)	0.35
GGT	30.17 (24.09–36.25)	29.02 (22.82–35.22)	0.79
Calcium	9.42 (9.23–9.60)	9.48 (9.35–9.61)	0.56
LDH	158.12 (149.54–166.69)	160.15 (152.53–167.77)	0.72
CRP	6.64 (1.26–12.03)	8.28 (3.92–12.64)	0.64
Total Bilirubin	0.61 (0.55–0.68)	0.50 (0.45–0.55)	0.01
PSA	13.07 (8.48–17.66)	11.33 (7.66–15.00)	0.56
PSA RATIO %	15.95 (14.16–17.74)	17.32 (13.59–21.05)	0.50
serum FLCk	23.61 (21.17–26.04)	29.58 (25.22–33.93)	0.02
serum FLCl	18.99 (16.49–21.50)	23.40 (16.80–30.00)	0.21
FLCr	1.27 (1.21–1.32)	1.39 (1.24–1.53)	0.13
Albumin	51.16 (48.92–53.40)	48.63 (45.95–51.30)	0.15
alfa1	3.38 (3.24–3.52)	3.67 (3.41–3.94)	0.06
alfa 2	8.35 (7.97–8.73)	9.11 (8.44–9.79)	0.05
beta 1	5.33 (5.08–5.58)	5.42 (5.02–5.82)	0.71
beta 2	4.21 (4.04–4.38)	4.17 (3.87–4.47)	0.81
gamma	11.61 (11.05–12.17)	12.40 (11.37–13.43)	0.18
testosterone	5.22 (4.79–5.66)	4.73 (4.11–5.35)	0.19
BMI	26.96 (25.83–28.08)	25.96 (23.91–28.01)	0.38
PT_RATIO	0.98 (0.95–1.00)	1.01 (0.96–1.06)	0.22
INR	0.98 (0.95–1.00)	0.99 (0.93–1.05)	0.57
APTT	31.42 (30.81–32.03)	31.65 (30.02–33.29)	0.79
APTT_RATIO	1.05 (1.03–1.07)	1.04 (0.98–1.11)	0.80
fibrinogen	338.28 (323.86–352.70)	397.76 (362.51–433.02)	0.00
NLR	2.44 (2.24–2.65)	3.12 (2.58–3.65)	0.02
MLR	0.32 (0.30–0.35)	0.36 (0.31–0.41)	0.14
PLR	165.18 (105.41–224.94)	151.14 (121.37–180.90)	0.68
A_G_ratio	1.05 (1.00–1.10)	1.10 (1.00–1.19)	0.37

**Table A2:** Age-stratified observed vs. expected MGUS (Kyle NEJM 2006) [34].

Age_Band	*n*	MGUS	Expected Prev	Expected	Obs Prev
50–59	12	4	0.017	0.2	0.3333333333333333
60–69	38	8	0.03	1.14	0.21052631578947367
70–79	54	14	0.05	2.7	0.25925925925925924
80+	14	9	0.075	1.05	0.6428571428571429

Overall SIR = 6.88 (95% CI 4.79–9.56).

**Table A3:** Associations of MGUS with prior malignancy and comorbidities.

Variable	Variable	MGUS Yes	MGUS No	Prevalence in Exposed %	*p* Value
Prior_malig	prior_malig	0	0	nan	1.0
Diabetes	dm	0	0	nan	1.0
High blood pressure	htn	1	5	16.7	0.6696
Cardio vascular desease	cvd	3	4	42.9	0.4332
Chronic Obstructive Pulmonary Disease	copd	2	1	66.7	0.2174
Liver pathology	liver	0	0	nan	1.0
Renal desease	ckd	1	0	100.0	0.3022
